# Evaluation of the community response to HIV and AIDS: Learning from a portfolio approach

**DOI:** 10.1080/09540121.2013.764395

**Published:** 2013-06-09

**Authors:** Rosalía Rodriguez-García, David Wilson, Nick York, Corinne Low, N'Della N'Jie, Rene Bonnel

**Affiliations:** a World Bank, Washington, District of Columbia, USA; b UK Department for International Development (DFID), Glasgow, UK; c School of International and Public Affairs, Columbia University, New York, NY, USA

**Keywords:** HIV, investing in communities, combination prevention, mixed method, evaluation, community-level results

## Abstract

While communities have played a large role in the HIV/AIDS response, their contributions and innovative approaches to HIV prevention, treatment, care and support have not always been the focus of systematic and rigorous evaluations. To address this gap, the World Bank led an evaluation of the impact of the community response to HIV, including country studies in Burkina Faso, India, Kenya, Lesotho, Nigeria, Senegal, South Africa and Zimbabwe over a three-year period. Due to the complexity and varied nature of community responses, the evaluation attempted to determine the results that investments have produced at the community level by applying a mixed method approach: Randomized Controlled Trials, quasi-experimental studies, qualitative studies and analytical studies including financial data. Specifically, the studies examined a typology of community response and the flow of funds to community-based organizations, while investigating the impact of the community responses on (1) knowledge and behavior, (2) use of services, (3) social transformation, and (4) HIV incidence. This editorial summarizes the results of this evaluation portfolio, finding that investments in communities have produced significant results, including, improved knowledge and behavior, and increased use of health services, and even decreased HIV incidence. Evidence on social transformation was more mixed, with community groups found to be effective only in some settings. Each study in the evaluation provides a partial view of how communities shape the local response; however, taken together they corroborate the common wisdom that communities can be a vital part of the global HIV/AIDS response.

## Overview

This editorial overview summarizes the key findings of the World Bank-led evaluation of the results achieved by community responses to HIV and AIDS, which includes 10 country-level evaluations in addition to overarching desk studies. We use the term ‘evaluation portfolio’ to refer to the set of 17 studies conducted as part of the evaluation. These studies provide the evidence for this overview of key findings. They are referred to as World Bank reports with country names and the year when the report was completed. A number of studies from this evaluation portfolio were selected for publication in this special issue of AIDS Care, and each provides details of their individual design, methodology, analytical approach, and findings.

Before the scale-up of the international response to the AIDS pandemic, communities in developing countries played a crucial role in providing services and care for those affected. This evaluation portfolio provides a comprehensive, mixed method evaluation of the effects of community responses to HIV and AIDS outcomes. The evaluation finds that under certain conditions, investments in community responses can be effective at increasing knowledge of HIV, promoting social empowerment for some issues, increasing access to and use of HIV services, and even decreasing HIV incidence – all through the effective mobilization of limited resources. This editorial details these findings and their supporting evidence, and then discusses potential policy actions and further research.

## Background: role of communities

Since the beginning of the Human Immunodeficiency Virus/Acquired Immune Deficiency Syndrome (HIV/AIDS) epidemic, communities have played an important role, often working in tandem with governments. Communities have been instrumental in developing innovative approaches to service up-take and delivery, and in accessing and empowering marginalized populations affected by the epidemic (Chillag et al., 2002). The first organizational responses came, almost universally, from affected individuals, their families and community groups. This HIV/AIDS response that comes from within the community is what we refer to as the “community response,” as described in Box 1. However, even though the focus of this evaluation is HIV/AIDS responses initiated, managed, or carried out by communities, that does not imply that such responses are always (or even typically) community funded. The donor investment in communities has been substantial. That investment has brought with it a mandate to better understand the effectiveness of community responses, and what role it can appropriately play going forward in the overall global response to the HIV/AIDS epidemic.

From 2003 to 2009, the four major HIV/AIDS donors combined – the UK's Department for International Development (DfID), the Global Fund to Fight AIDS, Tuberculosis and Malaria (the Global Fund), the US President's Emergency Plan for AIDS Relief (PEPFAR), and the World Bank – disbursed on average $690 million per year through civil society organizations, large and small (Bonnel et al., 2011). Alongside the resources, came the call to show results on these investments on the ground. The importance of rigorous evaluation of international aid activities, that would be closely linked to national policymaking, involve stakeholders, increase the knowledge base, and improve operations, became part of the global dialogue. Yet, many of the activities of civil society were not always the focus of rigorous evaluations. Where the activities of community-based organizations (CBOs) have been evaluated, it has necessarily been in an isolated fashion, often using qualitative tools (Foster, Makufa, Drew, Kambeu, & Saurombe, 1996; Seeley, Wagner, Mulemwa, Kengeya-Kayondo, & Mulder, 1991) or concentrated on measuring project or specific intervention impacts. Thus, the effects of community-based activities on the communities and population groups they serve remained largely unmeasured, even as international funding for the HIV/AIDS response scaled up, partly with the intention of supporting community groups (Kaiser Family Foundation, 2011).

Recognizing the need for a better understanding of community-level results of HIV/AIDS investments, in 2009 the World Bank and DfID, in partnership with the UK Consortium on AIDS and International Development, launched the multi-study evaluation reported in this special issue of AIDS Care with the intent to collect primary data and to promote the use of evaluative evidence for learning and innovation (York, 2012). The overarching question of the evaluation was: “What results has the community-based response to HIV/AIDS produced, and thus what return can be expected from future investments?” In this editorial article, we summarize the evaluation findings in four specific areas:

(1) Can community responses result in improved knowledge and behavior change?
(2) Can community responses increase access to and utilization of services?
(3) Can community responses cause meaningful social transformation and impact social norms, including stigma?
(4) Can these factors combine to decrease HIV incidence?

To provide insight into the inputs required to produce these results, this editorial also provides an overview of the flow of funds to communities and the allocation of funding by CBOs.

**Box 1: Definitions**

Communities can be described as:

- Sharing a *cultural identity* (members belong to a group that share common characteristics or interests), such as people living with HIV and AIDS, men who have sex with men, and sex workers.
- Sharing a *geographic sense of place* (a group in a location or an administrative entity). For instance, in Kenya, the National AIDS/STD Programme, Ministry of Medical Services defines a community as a collection of household units brought together by common interests, and/or made up of at least 5000 people (or 100 households) living in the same geographical area.

The *community response* can be defined as:

• The combination of actions and steps taken by communities, including the provision of goods and services, to prevent and/ or address a problem to bring about social change.
• *Community responses can be characterized* in six main ways:
  (1) types of implementing organizations and structures; (2) types of implemented activities or services and beneficiaries; (3) actors involved in and driving the response; (4) contextual factors influencing responses; (5) the extent of community involvement in the response; and (6) the extent of involvement of wider partnerships and collaborative efforts.

*Social transformation* is defined as:

the process by which society, organizations, and individual change happens, such as changes in behaviors or cultural norms and perceptions as a direct or indirect result of community action.

Source: Rodriguez-García et al. (2011).

## Methodological approach to the evaluation portfolio

**Figure 1.**
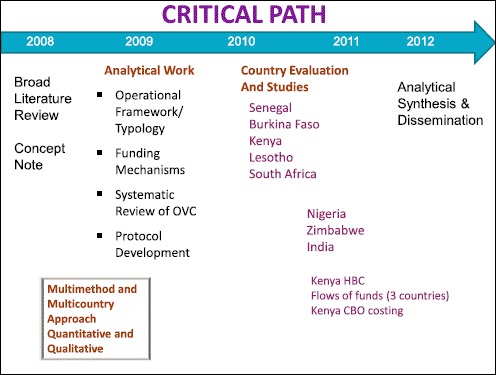
Design and implementation of the evaluation: A phase-in approach.

The design and implementation of this evaluation involved several phases that built on each other (see Figure 1). To address some of the methodological challenges and the highly contextual nature of community-level work, this evaluation applied a phase-in, mixed method approach with research designs, methods and instruments driven by the evaluation questions and country settings. It has resulted in a portfolio of 17 studies, including country-specific evaluations in Burkina Faso, India, Kenya, Lesotho, Nigeria, Senegal, South Africa, and Zimbabwe. These countries were selected because they were located in areas of high HIV prevalence, they allowed for the study of specific key interventions, and/or they addressed outcomes for at-risk and vulnerable populations. The same type of interventions was studied in several countries, and different sources of information were used to corroborate results. Triangulation of findings within the evaluation portfolio provided an additional level of analysis and supported learning.

The methodology of each study varied across countries as a function of the specific research questions to be studied and the respective context. Some country studies used an experimental design (Randomized Controlled Trials, or “RCTs”) with individual, household, or community randomization. Some studies were quasi-experimental, using longitudinal surveys or matching methods to establish comparison groups. The experimental and quasi-experimental studies used robust methods for establishing a “counterfactual,” meaning: What would have happened to a similar group of people in the absence of community-based interventions? Other studies used descriptive and analytical methods. Most country studies also collected a range of qualitative (social transformation) and financial data (flow of funds). Desk studies reviewed the existing documentation as well as new survey data to inform and complement the country evaluations. By using several methods, the limitations of any one particular method were mitigated (Adato, 2012; Rubio, 2012). Table 1 provides an overview of the focus and methodology of specific studies included in the evaluation portfolio. For a full explanation of methodologies used in specific studies, the accompanying articles in this journal provide further details.

**Table 1.** Evaluation portfolio: focus and methodologies by study.

Country evaluations	Focus	Method and analysis	Collected primary data?
Burkina Faso	Impact of community prevention activities on knowledge, prevention behavior, and stigma	Quasi-experimental: exposure to a national program as an instrumental variable for community group participation	Yes
India (Karnataka)	Impact of mobilization and empowerment among female sex workers	Quasi-experimental and qualitative: propensity score matching, multivariate regression, and case studies	Yes
India (Andhra Pradesh)	Impact of community collectivization among female sex workers and high-risk men	Multivariate regression, computation of odds ratios	Yes
Kenya	Understand funding and activities of CBOs and evaluate the impact of strong community response on knowledge, behavior, and service uptake	Quasi-experimental and qualitative: cluster propensity score matching and key informant interviews	Yes
Kenya (HBCT)	Ability to implement home-based testing in the presence of stigma and impact of testing effort on community leader and member stigma	Randomized controlled trial	Yes
Nigeria	Understand funding and activities of CBOs and evaluate the impact of strong community response on knowledge, behavior, and service uptake	Quasi-experimental and qualitative: cluster propensity score matching and key informant interviews	Yes
Nigeria	State-level secondary analysis to understand funding and activities of CBOs and evaluate the impact of strong community response on knowledge, behavior, and service uptake	Multivariate regression	No
Lesotho	Relationship between HIV/AIDS stigma and take-up of services/testing in a high prevalence area	Bivariate regression	No
Senegal	Impact of social mobilization on counseling and testing uptake (comparing peer mentoring to traditional sensitization)	Randomized controlled trial	Yes
South Africa	Impact of peer support and nutrition supplementation on treatment adherence	Randomized controlled trial	Yes
Zimbabwe	Impact of grass-roots community group membership on behavior, service utilization, and HIV incidence	Quasi-experimental: longitudinal data with individual fixed effects	Yes
Zimbabwe	Analysis of social spaces and critical dialog in HIV outcomes in Zimbabwe	Qualitative analysis: focus group discussions	Yes
Studies			
Typology of community response		Desk study	No
Cost structure of CBOs budgets in Kenya		Field study	No
Funding mechanisms		Survey and desk study	Yes
OVC review		Systematic review	No
CBOs resources and expenditures in Kenya, Nigeria, Zimbabwe		Field study	Yes

A consultative peer-review process was embedded in the evaluation at the global, national and local levels with experts, academics, partner organizations, civil society, and other stakeholders, to help ensure the rigor of the evaluation. Civil society consultations were facilitated by a purposeful partnership with the UK Consortium on AIDS and International Development (see www.aidsconsortium.org.uk for evaluation-related reports and publications).

## Findings from the evaluation portfolio

This section highlights some of the key findings from the evaluation portfolio. The evaluation found that while some donor funds reach communities, CBOs also rely heavily on volunteers and maximizing scarce resources to achieve impact. Prevention was found to be a large focus of CBO spending, and indeed the evaluation found impacts of CBO activity on HIV/AIDS prevention, including increases in knowledge of HIV avoidance (for example, knowing that condoms reduce HIV transmission in Kenya), reduction in high-risk behavior (for example, reduction of number of sexual partners in Zimbabwe and use of condoms by female sex workers (FSWs) in India). While most CBOs did not focus on directly delivering services themselves, CBOs can have an impact on take-up of services, which may increase service effectiveness – the evaluation found robust evidence of increases in service take-up (such as HIV testing or treatment adherence) in multiple countries. Evidence on social transformation (e.g., stigma reduction) was more mixed and not statistically significant. While community responses apparently contributed to social change among high-risk groups in Zimbabwe and India, in other settings, key informants believed that social transformation necessitated national policies that civil society could help to enforce. Perhaps most importantly, the evaluation found strong associative evidence that CBO activity can even decrease sexually-transmitted infection (STI) incidence, with a reduction in STIs found among FSWs participating in community organization in India, and a long-term reduction in HIV incidence found among women participating in community groups in Zimbabwe. Key evaluation findings are presented in more detail below, from inputs to outcomes along the logical results chain.

### Flow of funds: investment in communities

Communities have become much more involved in HIV and AIDS during the last decade, and the number of community organizations providing HIV/AIDS services has increased significantly. While a large portion of this is due to the growth of the epidemic itself, the rapid increase in donor funding during the last decade is another factor motivating community engagement. Analysis of a sample of 349 civil society organizations in 6 southern African studies showed that the average level of spending on HIV and AIDS was almost three times higher in 2005 than in 2001 (Birdsall & Kelly, 2007). Since then, further increases have taken place. For instance, the Global Fund reported that one-third of US$6.8 billion in country expenditures were implemented by CSOs and academia at the end of the 2009 reporting cycle (Global Fund, 2011).

In this evaluation, the flow of funds to CBOs and their uses were examined by performing three country case studies, in Kenya, Nigeria and Zimbabwe, and conducting a survey of 146 community organizations worldwide. The analysis of CBO budgets in Kenya and Nigeria shows that donor funding for HIV and AIDS is reaching small CBOs, and medium-size NGOs in Zimbabwe (World Bank, 2012). Financial assistance provided by bilateral and multilateral donors represented 33% of CBO budgets in Nigeria and 46% in Kenya (Idoko et al., 2011; Riehman et al., 2011).

**Figure 2.**
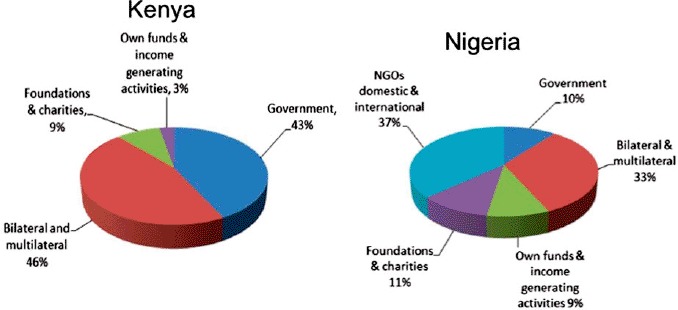
Channels mobilized by CBOs funding. Source: Riehman et al. (2011) and Idoko et al. (2011).

Figure 2 indicates that the resources mobilized by national funding channels, including governments, foundations, charities, and self-fundraising have become crucial sources of funding for CBOs. Nonetheless, CBOs operate with little total funding on average. For example, in Kenya CBOs had US$15,000 in annual funding, while in Nigeria they operated with US$17,000 on average (Idoko et al., 2011; Riehman et al., 2011).

Volunteers are a crucial resource for CBOs, perhaps allowing for the accomplishment of a greater CBO impact than their limited funding suggests. Unpaid volunteers alone add an estimated 56%, on average, to CBO budgets in Kenya, Nigeria, and Zimbabwe as shown in Table 2 (World Bank, 2012). The importance of volunteers – many of whom are caregivers – for CBOs suggests that these organizations are more sustainable over the long term than can be deduced by the high share of external resources in their funding.

**Table 2.** Value of unpaid volunteers as percentage of CBO/NGO budgets.

	Kenya	Nigeria	Zimbabwe
Number of volunteers per CBO/NGO	21	58	196
Value of unpaid volunteers’ free labour as percentage of CBO/NGO budgets	40%	48%	69%

Notes: CBO, community-based organization; NGO, nongovernmental organization.

**Figure 3.**
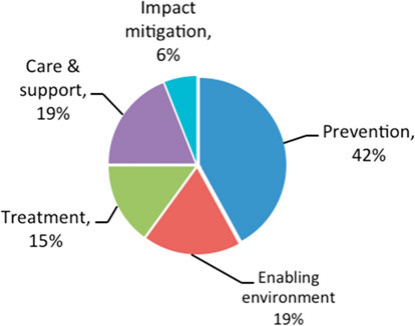
CBOs/NGOs expenditures by activities (percentage). Source: International HIV/AIDS Alliance in Bonnel et al. (2011).

Figure 3 shows the results of a worldwide survey that was carried out by the International HIV/AIDS Alliance as part of this evaluation. These CBOs (*n* = 146) were mainly small: two-thirds had fewer than 20 members, and they were mainly based in developing countries (89%) with a spread covering all regions. These data shed light on how CBOs use the resources they receive from the myriad funding channels discussed above. According to the survey, the largest share of their expenditures was for prevention. Within the prevention category, expenditures were the largest for high-risk groups, reflecting the comparative advantage that CBOs/NGOs have in reaching such groups.

In contrast to national HIV/AIDS programs, the surveyed organizations indicated that they spent only 15% on treatment, mainly for supporting people living with HIV and AIDS. Nearly 19% was spent on care and support, as well as on activities aimed at improving the enabling environment. The rest (6%) funded impact mitigation activities. Understanding the level of activity (based on resources available) and the budget allocations by CBOs provides important context for evaluating the impact of these organizations.

### Overview of community responses impacts

This evaluation portfolio found evidence that, depending on the country context, communities can have substantial impacts on knowledge and behavior, use of services, and even HIV incidence, with mixed evidence on social transformation.

Table 3 highlights some of the key findings of this evaluation portfolio. It outlines thematic areas and countries where evidence of effects was found, as well as the strength of the evidence. The strongest degree of evidence is provided by experimental studies (RCT) that yield causal evidence of impact. Quasi- experimental and longitudinal studies yield robust evidence with a lower strength, which we describe as “associative evidence.” Finally, there are cases where no statistically significant effect was found (labeled “null result”) or results from multiple tests found different results, which we label “mixed evidence.” In almost all cases, the evaluation found either positive or null results, and was not set up to test for negative results. However, in one area, stigma, both positive and negative impacts of community intervention were seen.

**Table 3.** Highlights of evidence concerning the effects of the community response to HIV/AIDS.

Activities	Outcome	Evidence source	Strength of evidence
Knowledge and behavior			
Information, awareness creation (speaking at public meetings, community theater, etc.)	Increased knowledge about HIV	Burkina Faso	Mixed evidence
		Kenya	Associative evidence
		Nigeria	Null result
Behavior change			
Promoting use of condoms	Increased condom use	Kenya, India (high risk)	Associative evidence
		Nigeria	Null result
Peer mentoring for HCT	Increased testing of HIV+ partner	Senegal	Causal evidence
Community group membership	Reduced risk behaviors	Zimbabwe, India (high risk)	Associative evidence
Services			
HIV counseling and testing (HCT)
Peer mentoring for HCT	Increased testing and pick up	Senegal	Causal evidence
Group membership (women)	Increased testing	Zimbabwe	Associative evidence
Promotion of HCT, mobile HCT	Increased testing	Kenya, Nigeria	Null result
Home-based HCT	Increased testing	Kenya	Causal evidence
Empowerment of FSW and MSM	Increased testing	India (high risk)	Associative evidence
Provision of PMTCT services	Increased use	Zimbabwe	Associative evidence
Prevention services and care	Increased use	Nigeria (rural areas)	Associative evidence*
ART peer support adherence and nutrition	Increased timeliness of clinic and hospital visits	South Africa	Causal evidence
Care and support			
Awareness of OVC rights	Increased awareness	Kenya	Associative evidence*
Provision of support to OVC	Increased services (rural areas)	Nigeria	Associative evidence*
Community group membership	Increased home-based care	Zimbabwe	Associative evidence
Income-generating activities and material support for PLWHA	Increased PLWHA support	Kenya, Nigeria	Null result
Social change/transformation			
Stigma	Reduced/increased stigma	Burkina Faso, Kenya, Lesotho, Nigeria, Zimbabwe	Mixed evidence (+/**—**)
Gender rights, violence	Gender violence and norms	Kenya, Nigeria	Mixed evidence
	Reduced police violence	India (high risk)	Associative evidence
Empowerment of groups at high risk of infection	Increased access/use of social rights	India (high risk)	Associative evidence
AIDS-health related outcomes			
Community group membership	Reduced HIV incidence	Zimbabwe	Associative evidence
Empowerment of FSW groups	Lower STI	India (high risk)	Associative evidence
Empowerment of MSM/Transgender	Lower STI	India (high risk)	Null result

Notes: AIDS, acquired immune deficiency syndrome; ART, antiretroviral therapy; HCT, HIV counseling and testing; HBCT, home-based counseling and testing; HIV, human immunodeficiency virus; OVC, orphans and vulnerable children; PLWHA, people living with HIV and AIDS; PMTCT, prevention of mother to child transmission; STI, sexually-transmitted infection; FSW, female sex workers; MSM/T, men who have sex with men/transgender individuals. (+/—) means both positive and negative impacts were found.

* For one sub-group only (e.g., rural areas).

### Impact on knowledge and behavior

**Figure 4.**
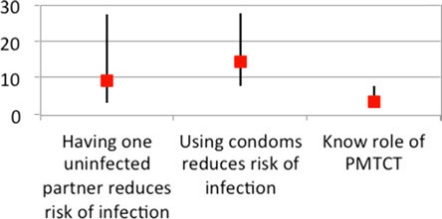
Strength of CBO engagement and HIV knowledge. Kenya 2011 (odds of increase).

The evaluation found that CBOs can impact both knowledge and behavior, but that the type of activity undertaken by CBO matters. Prevention activities by CBOs were found to have positive effects in some cases, but smaller impact in other situations. In Burkina Faso, community activities such as theater plays and radio debates were found to increase knowledge only partially, with men and women retaining differently. In Kenya, though, it was found that targeted community-level activities could lead to significant increases in knowledge of the benefits of monogamy and condom use (Figure 4). However, where knowledge is already high, CBOs may not have a significant effect, as was the case in Nigeria where 94% of surveyed households had already received mass-media messages about HIV, independent of CBO activities (Idoko et al., 2011; Riehman et al., 2011).

Knowledge interventions are often based on the idea that increased knowledge should impact behavior. Yet, there are many examples of communities and populations where despite high knowledge about HIV, little behavioral changes have taken place. Nonetheless, the evaluation found robust evidence of behavior change associated with CBO activity. In India, community group empowerment was associated with increased use of condoms among both FSWs and men who have sex with men or transgender (MSM/T) individuals. Associative evidence between the community response and condom use was also found in Kenya and Burkina Faso. In Zimbabwe, participation in a community group was associated with both increases in condom use and a reduction in the number of partners for women only, demonstrating that gender can be an important factor in behavior change. The types of community groups women participated in might have been more effective at altering behavior than those that men participated in, or women may be more open to risk reduction. The activities undertaken may have also been an important factor in Nigeria, where few community groups engaged in behavior change communication and the relationship between community groups and behavior was weak (De Walque, Kazianga, Over, & Rothenbuhler, 2011; Gregson, Nyamukapa, Sherr, & Campbell, 2011; Mohan et al., 2011; Riehman et al., 2011; Saggurti et al., 2012).

### Impact on use of health services

**Figure 5.**
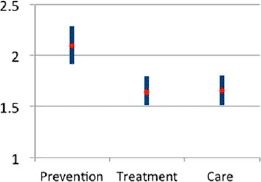
CBO density and service use in rural areas in Nigeria, 2011 (odds of utilization). Notes: CBO, community-based organization; diamond = odds ratio; line = 95% confidence interval.

Increasing use of health services is one area where the community response to HIV can strengthen the impact of other sources of support. Indeed, the evaluation found that a strong community response can cause greater use of existing HIV services, such as increased participation in prevention, treatment, care, and support in Nigeria, primarily in rural areas (Figure 5) (Idoko et al., 2011). In Zimbabwe, community group participation was found to increase both take-up of prevention of mother-to-child transmission and HIV counseling and testing (Gregson et al., 2011). In Senegal, an RCT yielded causal evidence that peer mentoring doubled the number of individuals participating in counseling and testing, and increased the number of HIV-positive individuals whose partners got tested (Arcand, Diallo, Slioune, Sakho, & Wagener, 2010).

**Figure 6.**
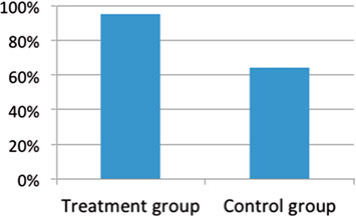
Percentage of individuals who have ever had an HIV test in Western Kenya due to HBCT. Notes: HBCT, home-based counseling and testing; HIV, Human Immunodeficiency Virus.

Community responses can also encourage treatment take-up. In South Africa, peer adherence support increased the timeliness of clinic visits in an RCT (Booysen, De Walque, Over, Hashimoto, & De Reuck, 2011). Evidence for high-risk groups in India confirms these results, with community empowerment increasing the use of government health services by FSWs. However, no such effect was found for men who have sex with men/transgender individuals, potentially indicating that stigma against this behavior by health workers represents too big a barrier for empowerment to overcome (Mohan et al., 2011; Saggurti et al., 2012). Encouraging results about increasing use of services in the presence of stigma were found in Kenya, however. There, an RCT on the effects of home-based counseling and testing found that even in the presence of stigma, whole-community testing efforts could be effective in getting individuals who had never had an HIV test to accept counseling and testing (Figure 6) (Goldstein, Low, Ndege, Pop-Eleches, & Thirumurthy, 2012). Finally, while in-country evaluations of orphans and vulnerable children (OVC) related results were not statistically significant, in a systematic review of community-based interventions’ effects on child outcomes, 86% of studies reviewed showed positive child outcomes (World Bank-OVC, 2010).

These results indicate that community responses can increase the demand for health services in the context of generalized and concentrated HIV epidemics among groups at high risk of infection. Dedicated support from community members and caregivers such as peer-mentoring is effective – more so than “less-personalized” approaches. However, the issue of stigma remains a major hurdle to increasing the use of prevention, treatment and care in general.

### Impact on social transformation

The evidence from the country evaluations including from the articles included in this journal issue indicates that there are complex pathways for community responses to contribute to social transformation that depend substantially on the population groups, country contexts, the geographic location, and the overall government policy. The evaluation found evidence that the community response can generate social changes among groups that are severely affected by the HIV epidemic. In India, for example, being a member of a sex worker community group was associated with access to social entitlements, reduced violence, and reduced police coercion (Mohan et al., 2011). Among Zimbabwe's general population, the community response led to significant changes in sexual risk perception and a reduction in stigmatizing attitudes toward people living with HIV and AIDS (Gregson et al., 2011).

However, national policies can make a big difference in the power of community groups to effect social change. For example, in India, although sex work is illegal, it is not a criminal offence, which opened the door to a dialogue with the police, which resulted in reduced police violence. In contrast, stigma attached to MSM/T and the existence of a repressive environment generally prevented MSM/T from accessing health services (Mohan et al., 2011; Saggurti et al., 2012). Furthermore, community members in Kenya and Nigeria believe that social changes related to gender norms and violence against women require a national policy shift. Communities can then follow up and help enforce those social policies. In Kenya, key informants perceived declines in violence against women as primarily linked to changes in national policies (such as the introduction of free primary-level education and the adoption of legislation protecting women from violence) (Riehman et al., 2011). In Nigeria, increased awareness, social consequences for the perpetrators, and the influence of government, NGOs, and other local organizations were often cited as reasons for the decline (Idoko et al., 2011) (see Table 4).

**Table 4.** Effects of community responses on selected social transformation indicators.

Country	Population	Catalyst	Results
Burkina Faso	Men	Deep-held cultural beliefs	• Low tolerance for HIV-infected people
India	Sex workers	Sex work is not a criminal offense	• Access to social entitlements • Decrease in violence • Decrease of police coercion
India	MSM/T	Discrimination persists	• Decrease access to services by MSM and transgender people
Kenya	General	National policy against household violence	• decrease in domestic abuse • decrease in violence against women
Kenya	Household	Home-based counseling and testing	• Decrease in stigma among community leaders • Increase in community stigma of HIV-infected people

As a note of caution, in the domain of stigma, some community HIV programs appear to carry the risk of unintended consequences. For instance, a negative but small (yet statistically significant) association was found in Burkina Faso between prevention programs and men's tolerance toward infected persons. A similar consequence resulted from home-based counseling and testing in Kenya. Home-based counseling and testing (HBCT) was found to lower the level of stigma of community leaders, but to raise the communities’ level of anger and disgust felt toward HIV-positive individuals (De Walque et al., 2011; Goldstein et al., 2012). This suggests that prevention programs could exacerbate personal stigmatizing attitudes by creating greater awareness of the disease. In this context, it is important to ascertain whether communities are equipped to address these deeply ingrained feelings in people. Qualitative approaches such as those based on community dialogue may prove adept at changing community member beliefs and practices.

### Impact on HIV incidence

Underlying most investment in HIV/AIDS programs is a hope that effective interventions can, eventually, bring a halt to the HIV epidemic. To do this, HIV incidence must be reduced. Although not all the country studies that make up this evaluation portfolio were able to measure changes in incidence, two of the studies explored this question, and provide associative evidence that community responses can affect incidence of HIV or STIs.

In Zimbabwe participation in a community group was associated with reduced HIV incidence for women during the period 1998–2003. Indeed, Zimbabwe is one of the few sub-Saharan African countries for which there is compelling evidence for a sustained decline in HIV prevalence driven by reduced levels of risk behavior (Gregson et al., 2010). In the following period (2003–2008), the decline in HIV incidence slowed, however. This study that is part of this evaluation portfolio used longitudinal data to control for individual characteristics, and thus tried to eliminate confounding factors from other factors than community group membership to single out the effects of the community response on HIV/AIDS, and thus offers strongly identified associative evidence of an impact of community group membership on HIV incidence.

In India, community group membership compared to non-membership was associated with lower prevalence of STIs, such as chlamydia and gonorrhea among FSWs (Mohan et al., 2011).

This evidence cannot be generalized to all groups and settings, but shows a promising suggestion that community response can play a role, along with other factors, in the overall battle to slow the spread of HIV.

## Discussion

This discussion is primarily based on quantitative data supported by qualitative findings of the 17 studies that comprised this evaluation portfolio. It also incorporates field observations, and key informant and expert contributions made during the consultative process of the evaluation at the local, national, and global levels.

This evaluation of the impacts of community responses found that the community response can be effective at combatting the HIV epidemic by improving knowledge and decreasing risk behavior, increasing access to and use of health services, sometimes contributing to social transformation, and even decreasing STI incidence in two cases. These findings are supported by and complement other major evaluations, such as the recent evaluation of the Avahan program (the India AIDS Initiative of the Bill and Melinda Gates Foundation) which found that participation, structural interventions, and organizational development activities coupled with access to services lead to improved outcomes (Rodriguez-García & Bonnel, 2012).

Although this evaluation found that community responses can have a large impact on the HIV epidemic, it should be noted that a community response cannot become a substitute for a national response. The clearest evidence of this is the findings around increasing use of services. In this area, community responses create a large impact by increasing take-up of services *provided by other actors.* Communities can help deliver specific results, as part of evidence-informed national implementation plans. Engagement with communities that keep these limitations in mind can help effectively utilize the advantages that communities *do* have, and avoid the trap of “doing a bit of everything with good intentions” to instead support “doing what can be done best with quality.”

A key implication of these findings is that evidence-informed policy and programming may be able to support community responses to achieve greater effectiveness by: (1) improving the targeting of services to the needs of the community; (2) creating better alignment of community-based activities with the HIV epidemic; and (3) strengthening the complementarity between community responses and national programs such as for HIV combination prevention measures. Program designers who are savvy about what CBOs and other community actors such as caregivers can realistically achieve can maximize the inputs and the results, while stakeholders can play a critical role in helping communities understand their epidemics and identify priorities for their catchment areas.

As part of supporting a strong community response, the amount and nature of the resources that flow to community groups need to be considered. At the present time, these resources are somewhat limited, and community groups “make do” largely with volunteer resources. If strengthening the community response itself is essential for longer-term impacts, sustainability, and greater effectiveness of national programs, then, alternative means of supporting community groups should be explored. This could mean: (1) exploring alternative modes of providing financial support to the local response, (2) strengthening national funding channels to facilitate access of community groups and small CBOs to funding – this would necessitate improving their technical capacity to collect and report data on expenditures, costs, budgets, and activities, (3) investing in well-defined rather than broad capacity strengthening for project staff and community groups, and (4) better understanding and support of caregivers in general and volunteers in particular who are a key resource for CBOs and communities at large.

Compensation for volunteers, many of whom are caregivers, varies hugely but can include stipends, social protection, in-kind payments and access to training and opportunities. The UK Consortium caution that unpaid work of these groups must not be seen as a cost saving or program efficiency. Programs need to assess and accommodate the type of compensation that would be appropriate to the community and programmatic context (UK Consortium on AIDS and International Development, 2012).

Undoubtedly, it is critical to optimize the investments to the global HIV/AIDS response so that resources are used efficiently and effectively, and there is still a dearth of evidence on the cost- effectiveness of funding community groups versus other methods of delivering the same impact. However, issues of efficiency and effectiveness need to be considered along with equity within the real context of where community groups work. Many do so in remote areas, working with disadvantaged, marginalized and hard-to-reach populations. Issues of equity and consideration of alternatives (“if not with this community mechanism, then with what?”) are equally important in the sense that providing services to hard- to-reach populations may involve higher unit costs than those incurred in delivering services to other groups or other geographic areas.

This evaluation also has implications for future research on community responses and their intended impact. One is that systematic evaluation of the community response may have more value than highly technical and complex but narrowly defined studies. A systematic approach would help establish a more continuous process of building knowledge about what works and what does not work, as well as to identify how to help shift investments to areas that would generate greater value for beneficiaries.

On the thematic front, there are several areas worth investigating further which are common across all community responses. For instance: (1) the evolution of local social capital and the role of volunteers (including how to sustain their commitment) and the continuum between non-compensated volunteers on the one hand, and fully-paid staff on the other, (2) the issues related to stigma and the role that real and perceived stigma plays in the access to and use of services by specific population groups, and (3) the programmatic pathways to achieving results at the community level ranging from inputs to impacts of key programs. The current approach is to develop program impact pathways as part of program design. Seldom do programs go back to review and update this results chain after the program has been implemented. Doing so, based on the empirical evidence and knowledge gained by implementing a particular program, would improve programming and results. Finally, it is clear that a value-for-money evaluation of community responses, compared to competing funding priorities, could help clarify in which areas communities are not only an effective mode of service delivery, but a “bargain” as well.

A final implication of this study is for research. The question drives the method, not the opposite. Researchers need to be able to select and apply the most appropriate research methods to examine different aspects of the community response taking into consideration the complexities of evaluating local responses. We have found that insights can be gleaned from multiple and different research methods, and that RCTs alone cannot illuminate all aspects of the community response. Creativity coupled with rigor is needed in the selection and application of research methods to examine community-based actions and activities.

## Conclusions

This evaluation portfolio provides robust evidence on the mostly positive contribution of community responses to national HIV and AIDS responses in many cases and circumstances. Nonetheless, there are limitations. This evaluation portfolio of country and desk studies do not provide a definitive answer to the effects of community responses on knowledge, behavior changes, use of HIV and AIDS services, social changes, and biological outcomes. Intervention-specific studies in selected community contexts would be helpful to corroborate and/or add robustness to the findings where this evaluation found mixed evidence, such as the role of perceived and real stigma in accessing and using services, or factors affecting treatment adherence.

Thus, the evaluation results, including those contained in the articles in this special issue of AIDS Care, do not support a one-size-fits-all design of community responses. However, the findings do indicate that global and national investments have produced results at the community level, which contribute to the outcomes of the global response to AIDS.

To conclude, taken individually, each study in this evaluation portfolio provides only a partial view of how investments in communities shape the local response to HIV and AIDS. However, when taken in the aggregate, this portfolio of 17 studies provides a robust body of evidence that supports the tenet that investing in communities achieves results that contribute to ameliorating the effects of and potentially halting the HIV epidemic.

The community response and community impact cannot be taken for granted, nor can it be guaranteed. A certain community fatigue could be looming on the horizon, triggered by ever-increasing needs, decreasing resources, and changing priorities. Policy-makers will need to find ways to engage and support communities, to ensure that the impacts shown in this evaluation continue to be a part of the global fight against HIV.
